# Evaluating Study Techniques for Australian Medical Students During Clinical Placement: A Scoping Review

**DOI:** 10.7759/cureus.103802

**Published:** 2026-02-17

**Authors:** Georgia Bartley, Samantha Waugh, Vinod Gopalan

**Affiliations:** 1 Medical Education, School of Medicine and Dentistry, Griffith University, Gold Coast, AUS

**Keywords:** clinical placement, medical students, post-pandemic, self-directed learning, study techniques

## Abstract

Transitioning from pre-clinical to clinical phases of medical education represents a unique challenge as students learn most content through self-directed learning (SDL), rather than the more prescriptive pre-clinical curriculum. There is a range of SDL study techniques employed by medical students on placement. The COVID-19 pandemic accelerated the adoption of digital resources, prompting a need to reassess the study techniques that best support clinical-year medical students. However, there is a lack of research on which study techniques Australian clinical-year medical students find most effective. The objective of this study is to evaluate the evidence on student-perceived utility of common study techniques for SDL whilst on clinical placement in Australia.

A qualitative scoping review of literature on PubMed and Medline Ovid was performed in 2024. Study inclusion criteria for articles were published articles, English language, publication within 20 years, and focus on clinical-year medical students. Exclusion criteria were review articles, investigations focusing on a specific educational intervention, and studies including only pre-clinical students. Studies were qualitatively appraised and synthesised by tabulation in Microsoft Excel (Microsoft Corp., Redmond, WA, US). Risk of bias analysis was not performed.

Nine studies from Australia, the USA, the UK, Thailand, Saudi Arabia, and Malaysia were analysed. This included seven cross-sectional, one mixed-methods, and one qualitative analysis. Sample size ranged from 12 to 350 students. Only two studies were conducted after the COVID-19 pandemic. Overall, the results of the included studies demonstrate a consistent trend towards third-party online tools, including question banks, mobile applications, and revision courses for SDL.

The strength of evidence on students' perceived efficacy of study techniques in the post-pandemic era presented is limited due to the small number of included studies and the lack of formal study appraisal. There is also poor generalisability of pre-pandemic and international studies to the contemporary Australian context. As there is a lack of a standardised tool to evaluate study technique utility, comparison between studies is difficult. Ongoing research is required to develop evidence-based guidelines that can assist students in commencing SDL whilst on clinical placement.

## Introduction and background

Medical education in Australia comprises 4-6-year degrees (both MD and MBBS), with pre-clinical and clinical medicine phases. Whilst Australia does not have a national medical licensing exam, comprehensive program evaluation by the Australian Medical Council standardises medical education. Problem-based learning forms the basis of the pre-clinical phase, with content delivered through lectures and workshops [1]. The clinical phase generally involves full-time placement at a metropolitan, regional, or rural hospital to learn applied medicine. On placement, learning outcomes are achieved through self-directed learning (SDL). SDL is the process through which students independently identify their own knowledge gaps and goals and develop suitable study techniques. As SDL encourages students to be dynamic and identify new learning goals, this approach is well-suited to the rapidly evolving nature of medical knowledge [2].

With the rise of personal electronic devices, a range of online medical education resources has become available for SDL, including student-made summary notes, question banks, and revision websites [3-5]. This has been accelerated in Australia by the COVID-19 pandemic, whereby isolation guidelines necessitated educational revolution from in-person delivery to flexible online study [6]. Strategies used to promote long-term retention of knowledge have also evolved. Literature indicates that more traditional study techniques, such as textbook re-reading and summarising, are inefficient and do not establish long-term knowledge retention [7,8]. Expanded-retrieval platforms (applications such as Anki (Ankitects Pty Ltd., New South Wales, Australia) that re-expose students to content and test recall at increasing time intervals) and practice knowledge application questions have been found to promote long-term knowledge retention [7]. Metacognition (being aware of one’s own cognitive processes and learning techniques) is also effective [8].

Transitioning from pre-clinical medicine can be challenging. Firstly, SDL requires learners to self-regulate the depth and breadth of study. Secondly, teaching in metropolitan hospitals has become difficult due to increased healthcare demands and patient complexity [1]. Consequently, students have reported the transition from pre-clinical to clinical medicine as a cause of stress [9]. Despite this, there is a lack of data on what study techniques Australian medical students find most useful for clinical medicine. When considering Kern’s six-step curriculum model, a model that identifies learning gaps, develops goals, and uses learning strategies to achieve these goals, it leaves students without evidence-based learning approaches that can aid SDL [10]. Further research is required to provide evidence-based study recommendations and improve educational outcomes for students struggling with the transition to clinical medicine. The objective of this study is to investigate the student-perceived utility of study techniques used during clinical medicine in Australia and globally. This will be achieved by performing a qualitative scoping review and appraising common study techniques. The review will follow PRISMA (Preferred Reporting Items for Systematic Reviews and Meta-Analyses) guidelines. This is done with the aim of providing evidence-based study approaches that can help students who are struggling achieve step 4 of Kern’s model and succeed in SDL.

## Review

The literature search was designed based on PRISMA scoping review guidelines. Due to the preliminary nature of this review, no protocol was registered.

Search strategy

An initial search was conducted on PubMed using the key terms ‘learning approaches’, ‘clinical placement’, and ‘medicine’ with Boolean operators. A list of search terms and operators is provided in Appendix 1.

Selection criteria

To maximise relevance to Australian clinical-year students, the inclusion criteria for articles were English language, published between 2004 and 2024, and focused on clinical-year medical students. Only published articles were included. As this investigation focused on the comparison of students' perceived efficacy of commonly used study techniques, review articles and studies of specific interventions were excluded. Studies including pre-clinical students were also excluded. The search was repeated in Medline Ovid.

Data extraction

Article abstracts were then screened for inclusion by a single author based on relevance to the study aims and methodological quality. No blinding or double-checking occurred. No authors were contacted for further sources. Formal appraisal of articles for study quality and risk of bias analysis was not performed. Due to the limited yield of studies, investigations of all methodologies and rigour were included. Data sought from the articles included the setting, participants, study methods analysed, and ranking of these techniques. Study limitations were also evaluated. These data were chelated and organised using Microsoft Excel (Microsoft Corp., Redmond, WA, US).

Appraisal

Article appraisal was qualitative, and no statistical analysis was performed. Studies were identified as ‘key articles’ - i.e., most relevant to the current Australian context - based on their geographical setting and publishing date relative to the COVID-19 pandemic. The study techniques highlighted by the literature were researched and qualitatively appraised.

**Figure 1:**
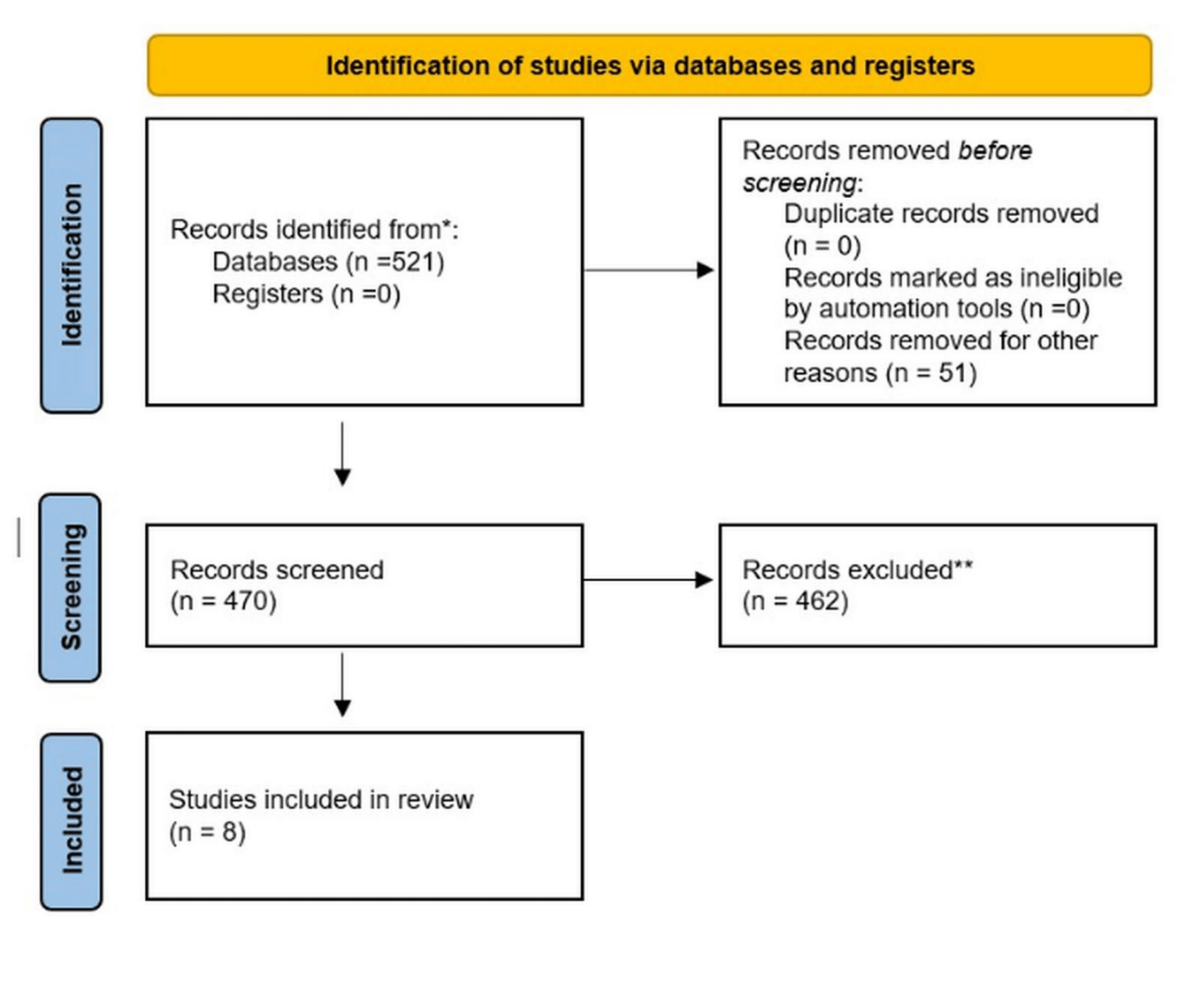
Flow diagram of the literature review process. Five hundred twenty-one papers were initially found, which were filtered down to 8 suitable papers. One additional paper was found during further research

As summarised in Figure 1, the initial PubMed search yielded 521 results. These were filtered by publishing date and language to 470 papers. Abstracts were then reviewed, and eight papers were selected as relevant to the key search terms. These studies were read in full and are summarised in Table 1 [4,5,11-16]. A Medline Ovid search yielded no additional relevant papers. An additional study identified through a further literature review was included as it was relevant to the study question but not captured in the initial search [17].

**Table 1:** Summary of key findings and limitations of the nine selected studies from the literature review *Cards refers to interactive case-based scenarios and questions. CD: compact disk; PBL: problem-based learning; GP: general practice; CBL: case-based learning; OSCE: Objective Structured Clinical Exam

Paper	Study setting	Participants	Resources investigated	Key findings	Limitations
Leong et al., 2007 [11]	International Medical University (IMU), University of Malaya (UM), Malaysia	305 clinical-year students (UM 165, IMU 140)	Learning resources (textbooks, lecture notes, pocketbooks, lecturers, the internet, guidelines, journals, CDs, and videos) and teaching (clinical teaching, lectures, and PBLs).	Textbooks followed by lecture notes and pocketbooks were the most utilised resources. Clinical teaching and lectures were the most useful style of learning experience. Students found that PBL was not useful for exam preparation.	Conducted prior to COVID-19 and the widespread availability of online learning. Different cultural environment to Australia. Does not discuss limitations and advantages of different resources, or how to aid transition into clinical practice.
Cooper and Elnicki, 2011 [12]	University of Pittsburgh, United States of America	130 year 3 students	Textbooks, review books, question books, and online resources (UpToDate).	Textbooks were preferred for exam preparation. Online resources such as UpToDate were used to prepare for ward rounds or admissions. Question book use was associated with better exam performance.	Conducted prior to COVID-19 and the rise of online learning. Focuses on how students can achieve the learning outcomes set by external medical colleges. This is less generalisable to Australia as placement is not guided by external learning outcomes or board examinations. Limited sample at a single teaching hospital.
Alzahrani and Alzahrani, 2012 [13]	King Abdulaziz University, Jeddah, Saudi Arabia	271 year 5 students, 205 year 6 students, and 71 interns	Assesses quality of learning sessions (lectures, tutorials, and clinical sessions), the quantity of sessions, methods of learning, and preferred sources of learning during surgical rotation.	Attendance at clinical sessions was rated as more important than lecture attendance, which progressively declined. Self-learning at home was the most preferred study technique. Preferred learning resources were faculty-assigned textbooks, followed by previous exams and summary notes. Non-faculty-assigned textbooks were the least preferred.	Different cultural environment to Australia. Conducted prior to COVID-19 and the rise of online learning. Only discusses learning during surgical placements. Discusses student uptake of lectures and how to make them more interactive, but there is limited discussion of the benefits/drawbacks of other study techniques such as past exams and textbook reading.
Baudains et al., 2013 [14]	Imperial College London, United Kingdom	12 year 5 students	Teaching sessions, websites/internet resources, textbooks, or reference books.	Textbooks (Oxford Handbook of Medicine), GP teaching, and Google were the most frequently discussed useful resources. Textbooks were the most popular.	Focuses only on resource usage during GP placement. Small sample of 12 students, using convenience sampling. Negative references were not recorded. Conducted prior to COVID-19 and the rise of online study resources.
Kantiwong and Charoensakulchai, 2018 [15]	Phramongkutaklao College of Medicine, Thailand	142 clinical-year students (years 4-6)	Interpersonal learning resources: senior doctors (faculties), residents, peers, or self-study.	Residents were the most favoured, followed by faculties then peers/self-study. Faculties were the most effective for stimulation of active learning. Self-study had the least impact on clinical skill acquisition.	Focuses on interpersonal relationships rather than online/textbook resources. Poor response rate from final-year students. Conducted prior to COVID-19 and the rise of online learning. Different cultural environment to Australia.
Jameel et al., 2019 [16]	King Abdulaziz University, Jeddah, Saudi Arabia	347 year 2, 3, and 4 students	Textbooks, online sources, medical websites, pocketbooks, online and print journal articles, lecture handouts, lecture notes, and test preparation textbooks.	80% of female and 29.2% of male students preferred textbooks. Online resources were used more by female than male students. The most favoured resources were lecture notes/handouts, online sources, and medical websites.	Different cultural climate to Australia with consideration of a language barrier to accessing textbooks. Conducted prior to COVID-19 and the rise of online learning. It is unclear if the analysis focuses on clinical-year students and if the survey was validated. Analysis mostly focuses on why students are not using textbooks and the associated barriers.
Wynter et al., 2019 [17]	University of New South Wales, University of Sydney, Australia	350 penultimate and final-year students	Assessed resources used for learning new content versus revision: small group tutorials, note-making, apps, online materials, question banks, online videos, medical literature, textbooks, watching lectures online, and attending lectures in person.	Question banks were the most popular resource overall. They were the most popular resource for revision, but also popular for learning new content. Lecture and tutorial attendance were the most popular resources for acquiring knowledge. Online resources were more popular than other resources. Age, gender, career aspiration, and previous degree completion did not relate to preferred learning resource.	Data were collected in 2015, prior to COVID-19 and the increased popularity of online study in medicine. No qualitative evaluation of student usage of materials.
Patel et al., 2024 [4]	University of Calgary, Canada	89 year 4 students	Self-directed: Cards*, online reading, podcasts, question banks, recorded lectures, student-made study guides, and videos. Direct teaching sessions.	Cards, then study guides, were the most highly rated self-directed resources. CBL was the most effective direct teaching session. Students used a mixture of both clerkship-provided resources and self-found resources.	Focus on both self-guided and faculty-run online learning, with additional focus on engagement strategies for direct teaching. OSCE study and aiding transition into clerkship are not discussed. Different medical education system due to the need to prepare for external examinations.
Baig et al., 2024 [5]	St George's University of London, United Kingdom	121 year 3 and 4 students (12 focus group participants)	Curriculum guides, clinical learning, school-run lectures, textbooks, and third-party resources (e.g., revision courses and medicine revision websites).	The school-provided core condition list was most preferred to guide learning. Third-party resources were preferred for self-study. Pre-clinical teaching guided clinical competence assessment preparation.	A major aim is to address concerns of board examinations causing disengagement from the medical school curriculum by improving curriculum guidance. Australia does not have a similar examination so there is limited generalisability.

Overview of studies

Seven cross-sectional studies [4,11-13,15-17], one mixed-methods study [5], and one qualitative analysis were reviewed [14]. Seven studies were published prior to the COVID-19 pandemic [11-17]. No studies addressed how students adapted study methods to clinical placement during the pandemic. Three papers were identified as most generalisable to contemporary Australia: Wynter et al. [17], Patel et al. [4], and Baig et al. [5].

Australian Study (Wynter et al.)

A cross-sectional survey of clinical-year medical students at the University of New South Wales and the University of Sydney assessed learning resources for new versus revision content [17]. Students rated 10 different study resources using Likert scales. Note-making and textbook reading were more common for learning new content, whilst question banks were the most popular for revision. Online medical education videos (92%) and online question banks (90.6%) were widely used, highlighting the role of digital resources even prior to COVID-19. However, the COVID-19 pandemic has significantly accelerated the adoption of online learning in medical schools, necessitating a re-evaluation of these study techniques [6]. Qualitative analysis was not performed. Further, the efficacy of study methods for Objective Structured Clinical Exams (OSCEs - a widely used practical exam for assessing clinical skills) was not elucidated. Thus, further research in Australian medical schools is required.

Canadian Study (Patel et al.)

This cross-sectional study evaluated the perceived utility of SDL and facilitator-directed learning tools during integrated online and in-person clerkships [4]. Students rated resources on Likert scales. Interactive case presentation ‘Cards’ was rated most useful, followed by student-made guides (e.g., Toronto Notes), whilst faculty-recorded lectures were least favoured [4,18,19]. These findings indicate a shift away from reliance on university-provided resources towards more self-directed and applied learning strategies. However, given the differences between Canadian medical education models and those in Australia, as well as the influence of Canada’s national accreditation examinations, the generalizability of these results is limited. Notably, the study does not explore OSCE preparation strategies or the transition from pre-clinical to clinical medicine (clerkship).

UK Study (Baig et al.)

A mixed-methods study assessed the use of curriculum guidance and study resources among clinical-year medical students [5]. Using a Likert scale, the study assessed students’ use and perceptions of curriculum guides, university-provided resources (e.g., lectures), and self-directed study approaches. Focus groups were also employed to explore student perspectives on the medical school curriculum [7]. Consistent with findings from Patel et al., third-party online resources, such as question banks and revision courses, were the most frequently used self-guided study tools, whereas textbooks were the least preferred [4,5]. These results suggest a clear shift away from traditional study methods towards more flexible, applied learning strategies. However, the study’s generalisability is limited by a low response rate (20%), and its emphasis on aligning university curricula with the UK national accreditation examination, which does not parallel the Australian system.

Other studies examining medical students' study techniques in different cultural contexts are summarised in Table 1 [11,13,15,16]. These include studies conducted in Malaysia, Saudi Arabia, Thailand, and the United States. Overall, these studies demonstrate a consistent trend away from traditional resources such as textbooks and pocketbooks towards third-party online tools, including question banks, mobile applications, and revision courses [4,5,17-19]. This shift was evident across multiple studies. However, the effectiveness of these methods on examination outcomes, including OSCE performance, remains unclear.

Common study techniques

To assist students in bridging the transition from pre-clinical to clinical medicine, evidence-based guidance on the utility of study techniques is needed. The present study appraises common techniques identified from the above literature. Other techniques that are useful in medical education include ‘flipped classrooms’ (a learning structure where students review material prior to class, then engage in discussions or exercises in class to deepen understanding) and team-based learning [20]. However, these are much more integral in the pre-clinical phase.

Active Learning Techniques

**Figure 2:**
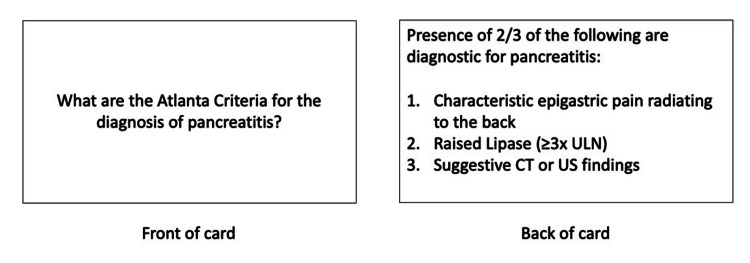
Example of a flashcard Sample flashcard to learn the criteria for the diagnosis of pancreatitis. This card can be inputted into the Anki program to be reviewed with spaced repetition. ULN: upper limit of normal; CT: computed tomography; US: ultrasound

Flashcards are a popular study technique grounded in the principles of active recall (retrieving previously learnt information in response to a question) and spaced repetition (reviewing information at progressively longer intervals) [21]. This can be done using programs like Anki to create and review digital flashcards, as demonstrated in Figure 2. Based on self-assessment of ease of recall, the program schedules subsequent reviews. Theoretically, with more reviews, recall of a card will improve, and thus, it can be re-reviewed at increasingly broad intervals. Flashcards can be created and shared between users, enabling students to focus their study on conceptually difficult topics or clinical knowledge from placement [22]. Literature supports the effectiveness of spaced repetition software like Anki in medical education for both students and trainees [8,21,23,24]. Anki is free software that can be accessed online or downloaded to personal devices, making it a flexible learning tool [22].

Anki can become time-consuming with the increasing number of daily flashcard reviews, so it may be unsustainable with placement hours [21]. Further, using pre-made flashcards can produce a 'parallel curriculum’, whereby students learn content that deviates from their university-specific curriculum [21]. Students should be made aware of this risk. Lastly, spaced repetition is less suitable for learning new information. Therefore, integrating flashcards with other study techniques is recommended [21]. This is consistent with Wynter et al., who found that note-making and attending lectures were the most preferred methods for acquiring new information [17].

**Figure 3:**
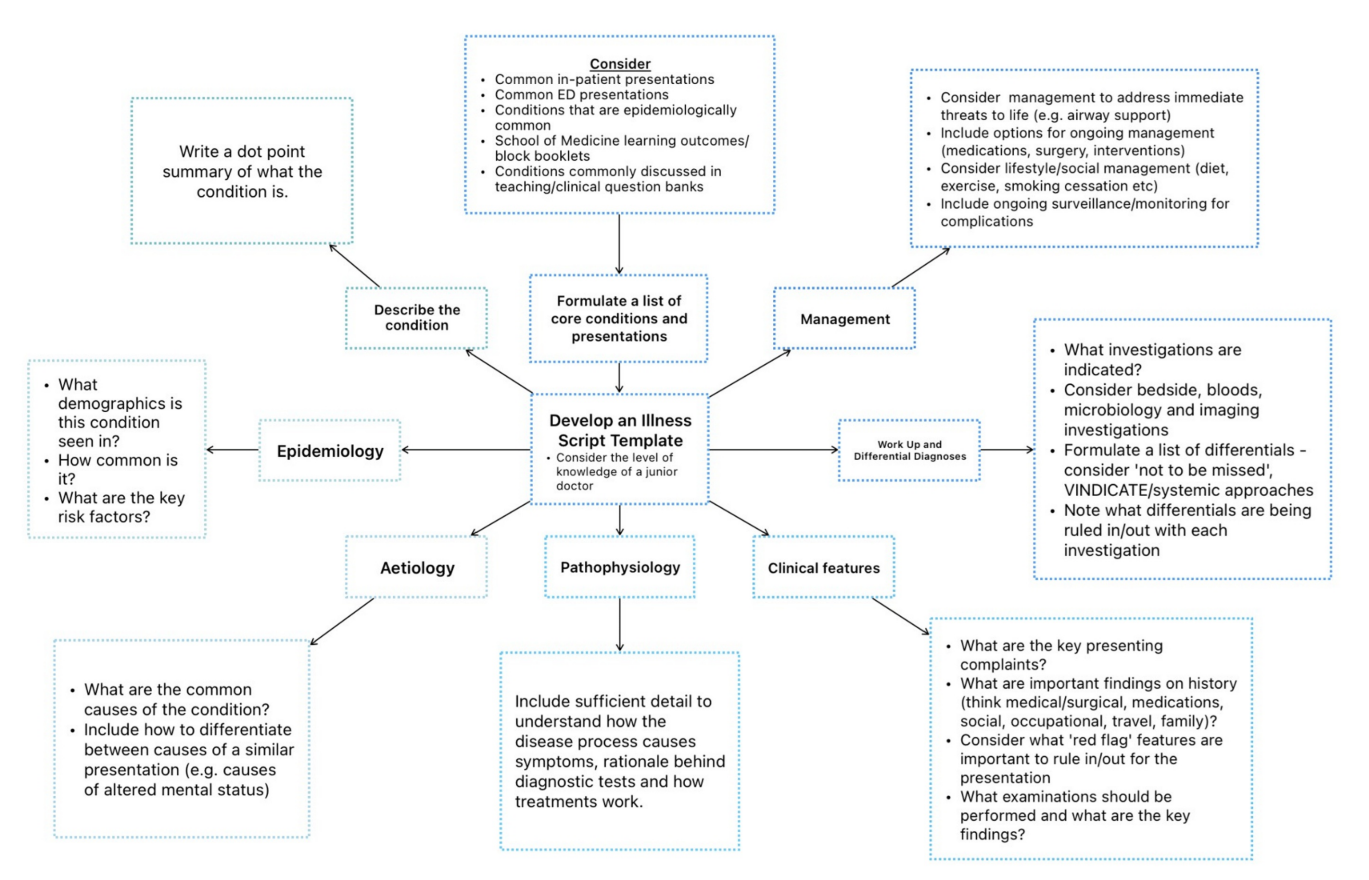
Sample illness script framework demonstrating a structured approach to understanding core features of a presenting condition. ED: emergency department

Illness scripts are organised summaries of a disease or clinical presentation that can be learnt and stored in memory. A typical illness script framework (shown in Figure 3) includes predisposing factors, pathophysiology, and clinical consequences of a condition [25]. As creating illness scripts involves critically synthesising resources, it offers an opportunity for in-depth learning, which is essential for mastering diagnostic and management concepts [25]. Furthermore, illness scripts can enhance clinical reasoning, as students can compare clinical presentations with defining features of a script to formulate differential diagnoses [25]. This makes illness scripts a powerful tool for developing both theoretical knowledge and diagnostic reasoning skills.

However, there are limitations. Students may struggle to identify key conditions to study and the depth of information to include. Further, summarising information is not an effective strategy for long-term knowledge retention. Thus, illness script writing may be best used in conjunction with spaced repetition techniques [8].

Special study modules (SSMs) are optional courses for students to learn an extension curriculum. This can encourage students to develop specialised medical interests or skills, as well as critical thinking [26,27]. SSMs have become a core component of medical education in the United Kingdom, covering academic, clinical, and professional competencies [27]. SSMs can be either arranged in a curriculum or delivered in protected time blocks [26]. Advantages of SSMs include opportunity for in-depth study, multidisciplinary learning, and the ability to address individual interests [26]. Their efficacy is highlighted by Ogut et al., who found that SSMs in cross-sectional anatomy improved medical students’ interpretation of cross-sectional imaging and ability to integrate knowledge into clinical practice [26]. However, making SSMs available across dispersed teaching hospitals could represent a barrier to implementation. This could be overcome by using e-learning online modules. E-learning is an approach that endeavours to use online material in a style that is collaborative but also flexible and asynchronous [28]. E-learning modules have a positive impact on all types of learners by improving students’ control over their learning process and supporting SDL [29].

Digital Tools

Databases, including point-of-care resources such as BMJ Best Practice (a British resource), UpToDate, and Therapeutic Guidelines (an Australian Guideline), can aid studies on placement [5,30,31]. Medical students have found UpToDate particularly useful for preparing for clinical placements [13]. Medical education websites such as AMBOSS, a clinical knowledge and question database aligned with the United States Medical Licensing Examination (USMLE), are also popular. These databases are useful as they provide accurate, accessible information, facilitating on-the-go study. However, these sources may not be consistent with Australian guidelines or university curriculum. Furthermore, whilst some databases are available through university libraries, subscription costs can pose a barrier to student access.

Online question banks are popular study resources that consist of multiple-choice questions (MCQs). Since MCQs form a large proportion of medical school examinations, question banks have been widely used to both learn new content and prepare for exams [32]. Knowledge application to clinical vignette-style questions can facilitate further learning and retention. Further, answer rationales can aid learning and help in identifying knowledge gaps [17]. Question banks are readily available on mobile devices via the internet or apps, allowing for flexible, opportunistic study [17].

Question bank usage has been linked to better performance on the USMLE Step 1 [33]. However, there are drawbacks: questions may not reflect the medical school curriculum, questions or rationales may be based on outdated guidelines or those that differ from local best practices, and many question banks require a paid subscription [32].

Artificial Intelligence (AI) is a concept that involves machines or computers making decisions, generating insights, learning from mistakes, or understanding language. AI has become more widely accessible and a powerful tool for SDL with the rise of applications such as ChatGPT [34,35]. ChatGPT is generative AI, meaning it has the ability to create content using information from internet resources [35]. A 2023 scoping review by Gordon et al. highlights the ways in which AI can be used for medical education. The main applications of AI to study proposed by Gordon et al. included using generative AI to process medical literature, generating summaries that can teach clinical management and assist with exam preparation, using AI platforms to create a personalised learning path, and enhancing anatomy and pathology education, e.g., training users to identify findings on images [34].

Thus, AI can assist students with SDL by generating learning objectives and plans, rapidly synthesising information, and providing answers to specific questions [35]. However, AI assistance should be employed with caution: AI can make mistakes, draw from poor-quality sources, or misinterpret incorrect information as factual. Students should apply critical thinking skills to appraise information produced by generative AI, especially in relation to evidence-based medicine [35].

OSCE Preparation

OSCEs are globally accepted, multistation examinations designed to evaluate students’ competence in various clinical skills and scenarios. They are incorporated into the assessment of both pre-clinical and clinical years of medical education [36]. Methods for OSCE preparation include revisiting clinical skills workshops from pre-clinical teaching, group practice, OSCE preparation websites (with both guides and practice scenarios), and formative OSCEs. Reflection on performance and SDL are associated with improved OSCE performance [37]. This can be facilitated by group practice. This practice often relies on OSCE scenarios and marking sheets from online resource banks, which typically require a subscription. Clinical placements also provide an opportunity to practice clinical skills. However, time constraints for the student to see the patient or patient frailty may limit the performance of a full ‘OSCE-style’ history or examination. Literature on OSCE preparation supports the importance of using past OSCEs, preparation guides from other students, and group study [38].

Future directions and limitations

With rising healthcare demands, illness-related placement disruptions, and the increasing complexity of hospitalised patients, clinical medicine students often face challenges in developing their knowledge, clinical skills, and soft skills during placements [1,39]. The discussed study techniques all have strengths and limitations. An example of how study techniques can be integrated and used for active learning on placement is provided in Appendix 2. From gaps identified through literature review, further research is required to improve the evidence base on which techniques are most effective so that evidence-based guidance can be given to students struggling to adapt to clinical medicine. In particular, further research investigating the utility of OSCE preparation techniques is required.

Whilst the present study provides insight into the current literature on study techniques used during clinical placement, there are methodological limitations. Firstly, there was no assessment of study quality or risk of bias analysis of the included studies, which reduces the quality of the literature review. Secondly, the review was performed by a single author over only two databases, introducing selection bias. The use of only a single author to interpret qualitative data also introduces the risk of biased interpretation of results from personal experience.

The review identified a clear gap in Australian medical education research between Wynter et al. and the present day. Due to its inclusion of international and pre-pandemic studies, the generalisability of this review to the modern Australian context is limited. Firstly, in non-English-speaking countries, language barriers can limit accessibility of resources such as textbooks, which was highlighted by Jameel et al. [16]. Furthermore, unlike Australia, some countries have external board examinations, in addition to university examinations. This introduces a separate curriculum that students have to study, which may inflate the perceived utility of resources such as question banks that target these exams. Further, as discussed earlier, COVID-19 triggered an educational revolution by swinging the delivery of university content from in-person to online [6]. This was accompanied by an increase in the use of internet resources for learning [40]. Thus, due to this change in learning preferences triggered by COVID-19, there is limited generalisability of evidence from studies conducted prior to the pandemic.

There are also overarching limitations carried by the included studies. Firstly, there is no uniform, validated outcome measure between studies, making it difficult to assess trends in perceived study technique utility between different groups and time periods. A direction for future research can include developing a validated tool for assessing students' perceived efficacy of study techniques to standardise the evidence base and assess trends. Secondly, the majority of included studies had a low response rate. Sampling bias may be introduced as the respondents are likely highly engaged students. This may reflect a difficult-to-access study population due to limited time whilst on clinical placement. Lastly, the included studies do not consider the impact of learning styles on perceived resource efficacy. A learning style is a student’s preferences for the learning process and learning conditions [41]. Learning styles play an important role in medical education as they can impact study hours and assessment scores [42]. Academic performance can be optimised by selecting resources that are cohesive with an individual’s learning type [43]. Therefore, to optimise SDL, students should be aware of their learning type and select resources that are most beneficial based on this. Learning styles, however, can evolve over time and can be multimodal. Thus, whilst learning styles can be used to guide resource selection, students should be encouraged to keep an open mind [44].

## Conclusions

The transition from pre-clinical to clinical medical education can represent a significant change in study pace, style, and setting, which can negatively impact students. Therefore, this study aimed to appraise the literature on study techniques during clinical placement and evaluate the strengths and limitations of common study techniques. From the available literature, there is an emerging trend away from traditional study techniques and towards online third-party resources such as question banks and flashcards. However, there is limited literature that is soundly generalisable to the contemporary Australian context. Thus, to provide evidence-based recommendations to students struggling with the transition to SDL in clinical medicine, further standardised investigations of perceived study technique utility are required.

## Figures and Tables

**Figure 4:**
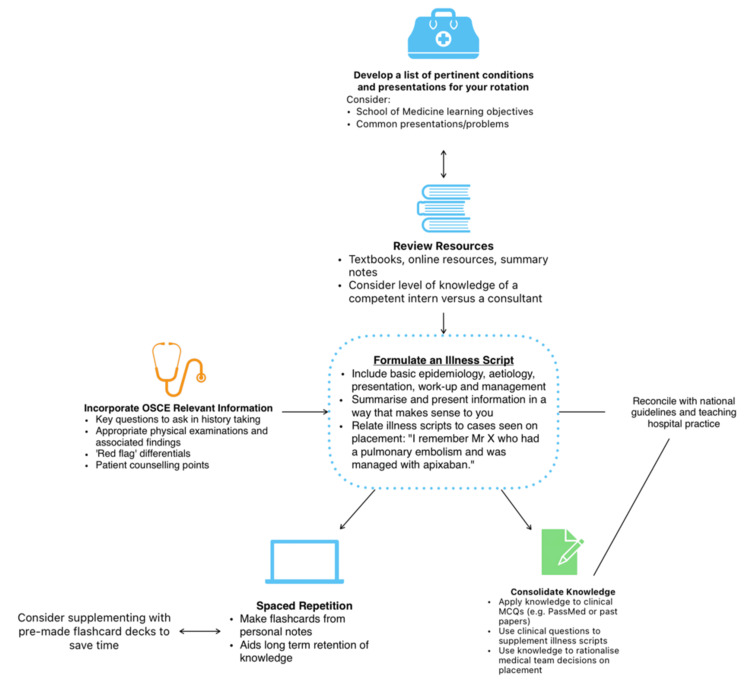
Graphical summary of common study techniques and how they can be implemented to complement each other in SDL The example models how students can integrate a range of study techniques for SDL and draw learning points from clinical placement. Figure developed by authors GB and VG based on the discussed literature review findings. MCQ: multiple-choice question; SDL: self-directed learning; OSCE: Objective Structured Clinical Exam

**Figure 5:**
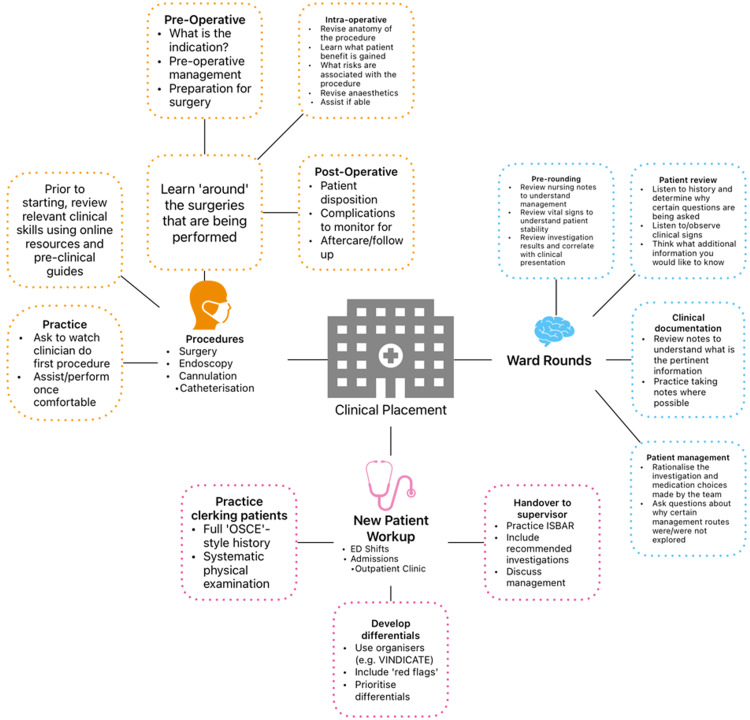
Graphical summary of potential learning opportunities whilst on clinical placement Figure developed by authors GB and VG based on the discussed literature review findings. OSCE: Objective Structured Clinical Exam; ED: emergency department; ISBAR: identify, situation, background, assessment, and recommendation

## Appendices

Appendix 1

**Table 2:** Search terms The above search terms were linked with Boolean operators to produce the following search phrase: (“Study technique*" OR "learning resource*" OR "education tool*") AND ("clinical medicine" OR Placement* OR "clinical year*" OR Clinical) AND (Medicine OR “med* school” OR “med* student*”) NOT (Pharmacy OR Nurs* OR "Allied Health" OR Physiotherap* OR "occupational therap*" OR Machine OR Vet*) The results were filtered to papers published in English between 2004 and 2024 (i.e., past 20 years at the time of review). Only published papers were included in the analysis.

Learning approaches	Clinical placement	Medicine	NOT
"Study technique*" OR "learning resource*" OR "education tool*"	"clinical medicine" OR Placement* OR "clinical year*" OR Clinical	Medicine OR “med* school” OR “med* student*”	Pharmacy OR Nurs* OR "Allied Health" OR Physiotherap* OR "occupational therap*" OR Machine OR Vet*

Appendix 2

Appendix 3** **
